# A multi-site study of the relationship between photoperiod and ovulation rate using Natural Cycles data

**DOI:** 10.1038/s41598-023-34940-z

**Published:** 2023-05-24

**Authors:** Divya Shanmugam, Matthew Espinosa, Jeffrey Gassen, Agathe van Lamsweerde, Jack T. Pearson, Eleonora Benhar, Sarah Hill

**Affiliations:** 1 Department of Electrical Engineering and Computer Science, Massachusetts Institute of Technology, Cambridge, MA 02139 USA; 2 Department of Psychology, Texas Christian University, 2955 S. University Dr., Fort Worth, TX 76129 USA; 3 Department of Anthropology, Baylor University, 1214 S. 4Th St., Waco, TX 76706 USA; 4 Natural Cycles USA Corp, New York, NY USA

**Keywords:** Behavioural ecology, Human behaviour

## Abstract

Many species exhibit seasonal patterns of breeding. Although humans can shield themselves from many season-related stressors, they appear to exhibit seasonal patterns of investment in reproductive function nonetheless, with levels of sex steroid hormones being highest during the spring and summer months. The current research builds on this work, examining the relationship between day length and ovarian function in two large samples of women using data from the Natural Cycles birth control application in each Sweden and the United States. We hypothesized that longer days would predict higher ovulation rates and sexual motivation. Results revealed that increasing day length duration predicts increased ovulation rate and sexual behavior, even while controlling for other relevant factors. Results suggest that day length may contribute to observed variance in women’s ovarian function and sexual desire.

## Introduction

In many habitats, environmental conditions vary considerably across seasons. These shifting conditions can present distinct survival and reproductive challenges to the organisms that inhabit them^1^. Winter months are characterized by lower temperatures, reductions in food availability, and increased risk for certain infectious diseases relative to summer months^2–5^. Such stressors demand investment in somatic maintenance, which often comes at the cost of investment in reproduction, particularly when energetic resources are limited^6–8^. Accordingly, many species exhibit breeding patterns that allow for the most costly expenditures in reproduction—such as those incurred from birthing and lactation—to occur during the summer months when exposure to environmental stressors is low^9–13^. Thus, for species with short (< 6 months; eg., goats and sheep^14^) or long (≥ 12 months; e.g., horses^15^) gestation periods, reproductive activity is highest in the summer months to allow conception and birthing to occur during months when an organism’s internal and external resources are abundant and favorable for gestation, birth, and lactation.

Although seasonal variability in reproductive function is well-established across sexes in a variety of non-human species—including rodents^16,17^, domestic cats and dogs^18,19^, and ring-tail lemurs^20^—much less is known about the impact of seasonal changes on human reproductive rhythms. Some initial evidence supports that human reproductive rhythms may be subject to seasonal variation. For example, research finds that levels of the sex hormone testosterone and investment in mate attraction and sexual behavior^21–24^ each peak in the spring and summer months. Others find that human conceptions peak in the spring and summer months when days are longer and reach a low point during the winter months when days are shorter^25,26^. These patterns are reasoned to occur in response to the changing environmental conditions in the spring and summer months, which are more favorable for embryonic development^26^ and implantation^27^. Indeed, these patterns are found to be robust to gradations in latitude^25,28,29^ and persist even after controlling for seasonal differences in sexual behavior^30^. Further, although the strength of seasonal influences on reproductive behavior varies across geographies^31^, populations^32^, and time^33^, they continue to be observed despite contraception allowing individuals to exert more control over reproductive timing based on the demands of the school calendar e.g.^4^, cyclical farming obligations e.g.^9^ and seasonal migratory employment e.g.^34^.

In the following, we seek to build on this prior work, examining whether human females exhibit seasonality in their reproductive function. Specifically, we used data collected from two large samples of women using the Natural Cycles fertility tracking application in each Sweden and the United States (U.S.) to examine the relationship between photoperiod—a reliably-occurring seasonal modulator of reproductive activity^6,25,30^—and (a) ovulation rates and (b) sexual motivation. We predicted that photoperiod would be positively related to women’s ovulation rate, with women ovulating more frequently when the days are longer. Additionally, we predicted that these patterns would be accompanied by complementary changes in women’s sexual motivation and behavior, with these outcomes also increasing when days are longer. Results seek to provide insight into human female reproductive function that have implications each for theory, clinical practice, and healthcare planning.

Unlike many species, contemporary humans are able to protect themselves from many of the energetic and immunological stressors associated with the fall and winter months. Such protections are a relatively new fixture in human evolutionary history, however. One of the legacies of these exposures is that many human biological systems—such as those that govern energy expenditure^35^, hormone release^36–39^, and immune function^12^—vary seasonally, similar to what is observed in seasonal breeders. For example, research finds powerful effects of photoperiod on multiple facets of immunological function, such as peripheral blood mononuclear cell proliferation^12^ which each peak in the winter months, when exposures to harsh environmental conditions would have historically been high^38,40,41^.

The season-based patterns in immune function and bodily repair are the opposite of the patterns observed for sexual function and reproduction, which peak in the spring and summer months. Despite the growing body of evidence suggesting seasonal patterns in testosterone production and conception rates, little is known about the impact of seasonality on human female ovarian function. Do women exhibit seasonal patterns of ovulation, similar to what is observed in other species of seasonally breeding organisms? Although most healthy, reproductive-aged women ovulate with a relatively high degree of regularity, research indicates that between 3.4% and 18.6% of these women’s cycles are anovulatory^42,43^. To date, factors that have been identified as contributors to this variability are almost exclusively person-based, such as differences in women’s obesity status^44^, history of anovulatory events^42^, fiber intake^45^, energy availability^22,37,46^, exercise intensity^47^, and exposure to stress^29,48^. Little attention has yet been given to the possibility that variability in human ovulation rates may also be accounted for by the changing seasons, with ovulation rates being higher as the days get longer.

The current study was designed to examine this possibility. Specifically, we use data collected from two large samples of women using the Natural Cycles fertility tracking application from each Sweden and the United States (U.S.). We limit our focus to Sweden and the U.S. because these are the two geographic samples for which we have regional information, which we then use to estimate photoperiod. The final sample included approximately 65,240 women with 353,411 reported cycles from the United States and 10,940 women with 72,498 reported cycles from Sweden. We predicted that longer days would correspond to a greater frequency of ovulatory cycles. Further, consistent with the idea that greater investment in female ovarian function in this context emerges in response to a larger prioritization of mating and reproductive goals during the spring and summer months, we predicted that (a) photoperiod would also predict increased sexual desire and behavior and (b) all patterns would persist while controlling for relevant biological factors (e.g., women’s menstrual cycle length), as well as secondary environmental factors (e.g., temperature) (Table 1).

**Table 1:** Sample characteristics split by sample.

		Sweden (n = 56,936)	United States (n = 326,137)
Age group	< 25	6739 (11.8%)	50,312 (15.4%)
	Between 25 and 30	21,453 (37.7%)	115,111 (35.3%)
	Between 30 and 35	19,869 (34.9%)	98,833 (30.3%)
	Between 35 and 40	6,421 (11.3%)	44,382 (13.6%)
	> 40	2,454 (4.3%)	17,499 (5.4%)
Parity	No	32,489 (72.7%)	182,487 (76.3%)
	Yes, once	6717 (15.0%)	28,836 (12.1%)
	Yes, twice	3234 (7.2%)	15,484 (6.5%)
	Yes, 3 times or more	2239 (5.0%)	12,454 (5.2%)
Education level	Elementary School	452 (1.0%)	475 (0.2%)
	High School	8795 (20.4%)	16,409 (7.5%)
	Doctoral Degree	678 (1.6%)	19,612 (8.9%)
	Trade/Technical/Vocational Training	4866 (11.3%)	16,197 (7.4%)
	University Degree	28,268 (65.6%)	167,555 (76.1%)
Relationship status	Single	4791 (12.9%)	17,811 (8.4%)
	It’s complicated	1326 (3.6%)	6460 (3.1%)
	In a relationship	23,792 (64.1%)	73,720 (34.9%)
	Engaged or married	7202 (19.4%)	113,334 (53.6%)

## Results

### Target model

#### Ovulation rate

In both samples (U.S. and Sweden), day length significantly predicted ovulation rate across multiple indicators of ovulation (Table 2). In the U.S. sample, results revealed that there was a significant positive relationship between day length and ovulation across all four measures of ovulation: Natural Cycles ovulation (*p* ≤ 0.001), Coverline ovulation (*p* ≤ 0.001), 3-over-6 ovulation (*p* ≤ 0.001), and LH tests (*p* ≤ 0.01), such that, women exhibited increased rates of ovulation as days grew longer. Three of these positive relationships hold with significance in the Swedish sample (Natural Cycles, *p* ≤ 0.001; Coverline, *p* ≤ 0.001; 3-over-6, *p* ≤ 0.001). Women in the Swedish sample instead exhibited a significant negative relationship between day length and ovulation as measured using LH tests (*p* ≤ 0.05).

**Table 2:** Target model inferential statistics for ovulation rate and sexual behavior measurements.

	3-over-6 Ovulation	Coverline Ovulation	LH Test Ovulation	Natural Cycles Ovulation	Logged Libido	Logged Sex
Covariate	*b* (*SE*)	*b* (*SE*)	*b* (*SE*)	*b* (*SE*)	*b* (*SE*)	*b* (*SE*)
Sweden (n = 56,936)						
Intercept	0.0004 (0.148)	0.0002 (0.093)	−1.19 × 10^–7^ (0.196)	0.001 (0.235)	0.686 ( 0.095)***	0.185 (0.014)***
Age	0.014 (0.003)***	0.006 (0.002)***	−1.36 × 10^–6^ (0.004)	0.024 (0.004)***	−0.006 (0.002)**	−0.001 (0.0003)***
BMI Proxy	0.009 (0.003)**	0.004 (0.002)*	−3.36 × 10^–6^ (0.004)	0.016 (0.005)**	0.003 (0.002)	0.0003 (0.0003)
Cycle Length	0.017 (0.003)***	0.005 (0.002)**	−1.64 × 10^–6^ (0.004)	0.023 (0.004)***	−0.005 (0.002)**	−0.001 (0.0002)***
Day Length	0.0003 (0.00004)***	0.0004 (0.00003)***	−1.26 × 10^–4^ (0.00005)*	0.001 (0.00006)***	0.00002 (0.00002)	0.00001 (0.000003)***
United States (n = 326,137)						
Intercept	0.0001 (0.064)	0.000003 (0.071)	8.25 × 10^–7^ (0.087)	0.0004 (0.144)	0.640 (0.040)***	0.242 (0.006)***
Age	0.003 (0.001)***	0.0001 (0.001)	7.75 × 10^–5^ (0.001)	0.011 (0.003)***	−0.005 (0.001)***	−0.002 (0.0001)***
BMI Proxy	0.002 (0.001)	0.0001 (0.001)	1.61 × 10^–5^ (0.001)	0.008 (0.003)*	0.003 (0.001)***	−0.0001 (0.0001)
Cycle Length	0.007 (0.001)***	0.0001(0.001)	6.83 × 10^–5^ (0.002)	0.013 (0.002)***	−0.006 (0.001)***	−0.001 (0.0001)***
Day Length	0.001 (0.00004)***	0.002 (0.00004)***	1.56 × 10^–4^ (0.00005)**	0.004 (0.00009)***	0.0001 (0.00002)***	0.00002 (0.000003)***

Day Length = number of minutes of ambient day light; BMI = body mass index; ***p ≤ .001; **p ≤ .01; *p ≤ .05.

#### Logged libido and sexual behavior

In addition to examining the relationship between day length and ovulation rate, we also assessed whether day length was associated with corresponding effects on women’s logged libido and sexual behavior frequency (see Table 2). For these analyses, we utilize a generalized Gaussian mixed-effects model, as logged libido and logged sexual behavior are continuous, cycle-specific variables. Results showed that across both geographic samples, day length was a significant positive predictor of sexual behavior (*p* ≤ 0.001). Studying the relationship between day length and logged libido, we found a significant positive association in the U.S. sample (*p* ≤ 0.001) and a nonsignificant relationship in the Swedish sample (*p* > 0.10).

### Follow-up models

#### Do the results of the target models hold when controlling for temperature?

When controlling for the daily temperature of each individuals’ central logged location, the relationship between day length and ovulation rate persisted (see Table 3). That is, day length remained a significant positive predictor of ovulation rate across the three temperature-based measures of ovulation (i.e., Natural Cycles algorithm, Coverline, and 3-over-6; *p*s ≤ 0.009). While controlling for daily temperature, day length instead is not a significant predictor of LH test measured ovulation rate (U.S., *p* = 0.084, and Sweden, *p* = 0.066), however the trend of these results is consistent with results of the other ovulation rate measures, with longer days being associated with increased rates of ovulation.

**Table 3:** Temperature-controlled model inferential statistics for ovulation rate and sexual behavior measurements.

	3-over-6 Ovulation	Coverline Ovulation	LH Test Ovulation	Natural Cycles Ovulation	Logged Libido	Logged Sex
Covariate	*b* (*SE*)	*b* (*SE*)	*b* (*SE*)	*b* (*SE*)	*b* (*SE*)	*b* (*SE*)
SW (n = 56,936)						
Intercept	0.0004 (0.149)	0.0002 (0.094)	−1.18 × 10^–7^ (0.198)	0.0007 (0.239)	0.669 (0.096)***	0.180 (0.014)***
Age	0.013 (0.003)***	0.006 (0.002)***	−1.34 × 10^–6^ (0.004)	0.022 (0.005)***	−0.006 (0.002)**	−0.001 (0.0003)***
BMI Proxy	0.009 0.003)**	0.004 (0.002)*	−3.35 × 10^–6^ (0.004)	0.015 (0.005)**	0.003 (0.002)	0.0003 (0.0003)
Cycle Length	0.016 (0.003)***	0.005 (0.002)*	−1.62 × 10^–6^ (0.004)	0.022 (0.004)***	−0.005 (0.002)**	−0.001 (0.0002)***
Day Length	0.0002 (0.00006)***	0.0003 (0.00004)***	−1.25 × 10^–4^ (0.00008)	0.0003 (0.00009)**	−0.00002 (0.00003)	−0.000003 (0.000004)
Temperature	0.004 (0.001)***	0.002 (0.0008)**	−1.04 × 10^–5^ (0.001)	0.008 (0.002)***	0.001 (0.001)	0.0003 (0.00007)***
United States (n = 326,137)						
Intercept	0.0001 (0.064)	0.000003 (0.072)	8.42 × 10^–7^ (0.087)	0.0003 (0.144)	0.640 (0.040)***	0.242 (0.006)***
Age	0.003 (0.001)***	0.0001 (0.001)	7.95 × 10^–5^ (0.001)	0.011 (0.003)***	−0.005 (0.001)***	−0.002 (0.0001)***
BMI Proxy	0.002 (0.001)	0.00007 (0.001)	1.64 × 10^–5^ (0.001)	0.008 (0.003)*	0.003 (0.0008)***	−0.0001 (0.0001)
Cycle Length	0.007 (0.001)***	0.00009 (0.001)	7.01 × 10^–5^ (0.002)	0.013 (0.002)***	−0.006 (0.0007)***	−0.001 (0.0001)***
Day Length	0.001 (0.00005)***	0.002 (0.00005)***	1.54 × 10^–4^ (0.00007)*	0.004 (0.0001)***	0.0001 (0.00003)***	0.00001 (0.000004)***
Temperature	−0.0006 (0.0004)	0.0002 (0.0004)	1.21 × 10^–5^ (0.005)	−0.003 (0.0007)***	−0.001 (0.0002)*	0.000004 (0.00003)

Day Length = number of minutes of ambient day light; BMI = body mass index; *** *p* ≤ .001; ** *p* ≤ .01; *p ≤ .05.

Similarly, we examined the impact of day length on logged libido and sexual behavior in the US sample and found that day length remained a significant positive predictor of both logged sexual behavior frequency and libido (*p* ≤ 0.001). However, these relationships became nonsignificant in the Swedish sample (*ps* ≥ 0.40; see Table 3)*.*

#### Do the results hold when we restrict our results to participants likely to be fertile (ages 18–45)

Further restricting analyses to women between 18 and 45 years old, the positive association between day length and ovulation rate remained significant across the three basal body temperature measures of ovulation rate (i.e., Natural Cycles algorithm, Coverline, and 3-Over-6) in both U.S. and Sweden samples (*p*s ≤ 0.01) (Table 4). As with the prior alternative models, day length was not significantly associated with LH test measured ovulation rate in the Sweden samples, (*p* = 0.12) and a significant positive predictor in the US sample (*p* = 0.02), when restricting analyses to women between 18 and 45 years old. In the U.S. sample, day length remained a significant positive predictor of both logged sexual behavior (*p* ≤ 0.001) and libido (*p* ≤ 0.001). As with the earlier analyses, we found both relationships to be nonsignificant in the Swedish sample.

**Table 4:** Age-controlled model inferential statistics for ovulation rate and sexual behavior measurements.

	3-over-6 Ovulation	Coverline Ovulation	LH Test Ovulation	Natural Cycles Ovulation	Logged Libido	Logged Sex
Covariate	*b* (*SE*)	*b* (*SE*)	*b* (*SE*)	*b* (*SE*)	*b* (*SE*)	*b* (*SE*)
Sweden (n = 56,936)						
Intercept	0.0004 (0.160)	0.0002 (0.109)	−7.60 × 10^–8^ (0.209)	0.0007 (0.238)	0.757 (0.103)***	0.181 (0.015)***
Age	0.013 (0.003)***	0.005 (0.002)**	1.41 × 10^–6^ (0.004)	0.021 (0.005)***	−0.009 (0.002)***	−0.001 (0.0003)***
BMI Proxy	0.008 (0.003)*	0.004 (0.002)	−2.65 × 10^–6^ (0.004)	0.014 (0.005)**	0.003 (0.002)	3.64 × 10^–4^ (0.003)
Cycle Length	0.015 (0.003)***	0.005 (0.0002)*	6.52 × 10^–7^ (0.004)	0.021 (0.004)***	−0.005 (0.002)**	−0.001 (0.002)***
Day Length	0.0003 (0.00006)***	0.0004 (0.00005)***	−1.24 × 10^–4^ (0.00008)	0.0003 (0.0001)**	−0.000007 (0.00004)	−6.04 × 10^–7^ (0.000004)
Temperature	0.004 (0.001)***	0.002 (0.0009)*	−1.04 × 10^–5^ (0.001)	0.008 (0.002)***	0.0008 (0.0006)	2.96 × 10^–4^ (0.00008)***
United States (n = 326,137)						
Intercept	0.000004 (0.069)	0.000003 (0.076)	4.13 × 10^–7^ (0.093)	0.0003 (0.157)	0.680 (0.042)***	0.251 (0.006)***
Age	0.0001 (0.001)	0.00009 (0.001)	2.52 × 10^–5^ (0.002)	0.010 (0.003)***	−0.006 (0.0009)***	−0.002 (0.0001)***
BMI Proxy	0.00009 (0.001)	0.00007 (0.001)	9.57 × 10^–6^ (0.001)	0.007 (0.003)*	0.003 (0.0008)***	−2.59 × 10^–4^ (0.0001)*
Cycle Length	0.0001 (0.001)	0.00008 (0.001)	2.23 × 10^–5^ (0.002)	0.011 (0.003)***	−0.006 (0.0007)***	−0.001 (0.0001)***
Day Length	0.002 (0.00006)***	0.002 (0.00006)***	1.63 × 10^–4^ (0.00008)*	0.005 (0.0001)***	0.0001 (0.00003)***	1.25 × 10^–5^ (0.000004)**
Temperature	0.0001 (0.004)	0.0002 (0.0004)	1.20 × 10^–5^ (0.0005)	−0.003 (0.001)**	−0.0005 (0.0002)*	−8.19 × 10^–6^ (0.00003)

Day Length = number of minutes of ambient day light; BMI = body mass index; *** *p* ≤ .001; ** *p* ≤ .01; *p ≤ .05.

#### Influence of equinox on ovulation rate

We find *R*^2^ scores of < 0.05 when we apply peak fitting approaches, and that the difference between ovulation rates for days with 12 h are not significantly different from ovulation rates for days with approximately equivalent day length and night length (day length measured to be between 11 and 13 h), and all other days (*ps* > *0.12)*. When considering logged libido and logged sex as dependent variables, we find similar results in each geographical cohort (ps > 0.4 in both analyses). Therefore, the results suggest that there are no significant effects of spring or autumn equinoxes on the relationship between day length and ovulation rate, logged libido, and sexual behavior.

### Robustness to range normalization

GPBoost relies on *gradient descent* to identify model parameters. Gradient descent is known to be sensitive to the scale of different covariates. Here, we sought to replicate the earlier experiments on range-standardized data while standardizing the measure of day length to values between 0 and 48, corresponding to units of half hours. This closely reflects the ranges of the other covariates: age (17–64), BMI (13–56), and cycle length (15–40).

Although the model fit is not significantly different (as measured by log likelihood on a held-out test set), the observed pattern of results differed somewhat (Tables 5, 6, 7). Specifically, all reported results remained significant in the US cohort (with the exception of LH test results, discussed below), but we found fewer significant relationships between day length and ovulation in the Swedish cohort.

**Table 5:** Range-standardized target model inferential statistics for ovulation rate and sexual behavior measurements.

	3-over-6 Ovulation	Coverline Ovulation	LH Test Ovulation	Natural Cycles Ovulation	Logged Libido	Logged Sex
Covariate	*b* (*SE*)	*b* (*SE*)	*b* (*SE*)	*b* (*SE*)	*b* (*SE*)	*b* (*SE*)
Sweden (n = 56,936)						
Intercept	0.00002 (0.148)	0.001 (0.093)	−0.001 (0.196)	0.003 (0.374)	0.686 (0.095)***	0.185 (0.014)***
Age	0.009 (0.003)**	0.015 (0.002)***	0.007 (0.004)	0.047 (0.008)***	−0.006 (0.002)**	−0.001 (0.0003)***
BMI Proxy	−0.012 (0.003)***	0.003 (0.002)	−0.012 (0.004)	0.024 (0.010)*	0.003 (0.002)	0.0002 (0.003)
Cycle Length	0.048 (0.003)***	0.007 (0.002)***	0.004 (0.004)	0.065 (0.005)***	−0.005 (0.002)**	−0.001 (0.002)***
Day Length	0.004 (0.001)**	0.001 (0.001)	−0.006 (0.002)	0.0002 (0.002)	0.0005 (0.001)	0.0003 (0.001)***
United States (n = 326,137)						
Intercept	0.001 (0.067)	0.001 (0.048)	−0.001 (0.087)	0.003 (0.165)	0.640 (0.040)***	0.242 (0.006)***
Age	0.003 (0.001)**	0.012 (0.001)***	0.008 (0.001)	0.039 (0.003)***	−0.005 (0.001)***	−0.002 (0.0001)***
BMI Proxy	−0.012 (0.001)***	−0.002 (0.001)**	−0.007 ()0.001	0.015 (0.004)***	0.003 (0.001)***	−0.0001 (0.0001)
Cycle Length	0.056 (0.001)***	0.007 (0.001)***	0.009 (0.002)	0.071 (0.003)***	−0.006 (0.001)***	−0.001 (0.0001)***
Day Length	0.003 (0.001)*	0.008 (0.001)***	−0.008 (0.002)	0.018 (0.003)***	0.003 (0.001)***	0.0004 (0.0001)***

Day Length = number of minutes of ambient day light; BMI = body mass index; *** *p* ≤ .001; ** *p* ≤ .01; *p ≤ .05.

**Table 6:** Range-standardized temperature-controlled model inferential statistics for ovulation rate and sexual behavior measurements.

	3-over-6 Ovulation	Coverline Ovulation	LH Test Ovulation	Natural Cycles Ovulation	Logged Libido	Logged Sex
Covariate	*b* (*SE*)	*b* (*SE*)	*b* (*SE*)	*b* (*SE*)	*b* (*SE*)	*b* (*SE*)
Sweden (n = 56,936)						
Intercept	0.0001 (0.147)	0.001 (0.106)	−0.0005 (0.197)	0.002 (0.378)	0.669 (0.096)***	0.180 (0.014)***
Age	0.009 (0.003)***	0.016 (0.002)***	0.008 (0.004)*	0.048 (0.008)***	−0.006 (0.002)**	−0.001 (0.0003)***
BMI Proxy	−0.011 (0.003)***	0.004 (0.004	−0.010 (0.004)**	0.025 (0.010)**	0.003 (0.002)	0.0003 (0.0003)
Cycle Length	0.047 (0.003)***	0.007 (0.007)**	0.005 (0.004)	0.064 (0.005)***	−0.005 (0.002)**	−0.001 (0.0002)***
Day Length	0.007 (0.002)***	0.001 (0.001)	−0.0001 (0.002)	0.001 (0.003)	−0.001 (0.001)	−0.0001 (0.0001)
Temperature	−0.002 (0.001)*	−0.0003 (0.001)	−0.005 (0.001)***	−0.001 (0.002)	0.001 (0.001)	0.0003 (0.0001)***
United States (n = 326,137)						
Intercept	0.001 (0.065)	0.001 (0.048)	−0.0004 (0.087)	0.003 (0.156)	0.640 (0.040)***	0.242 (0.006)***
Age	0.003 (0.001)**	0.012 (0.001)***	0.007 (0.001)***	0.038 (0.003)***	−0.005 (0.001)***	−0.002 (0.0001)***
BMI Proxy	−0.012 (0.001)***	−0.001 (0.001)	−0.006 (0.001)***	0.012 (0.003)***	0.003 (0.001)***	−0.0001 (0.0001)
Cycle Length	0.054 (0.001)***	0.007 (0.001)***	0.008 (0.002)***	0.068 (0.003)***	−0.006 (0.001)***	−0.001 (0.0001)***
Day Length	0.003 (0.002)*	0.006 (0.001)***	−0.006 (0.002)**	0.018 (0.003)***	0.004 (0.001)***	0.0004 (0.0001)***
Temperature	−0.0002 (0.0004)	0.0003 (0.003)	−0.0004 (0.005)	0.0001 (0.001)	−0.001 (0.0002)*	0.000004 (0.00003)

Day Length = number of minutes of ambient day light; BMI = body mass index; *** *p* ≤ .001; ** *p* ≤ .01; *p ≤ .05.

**Table 7:** Range-standardized age-controlled model inferential statistics for ovulation rate and sexual behavior measurements.

	3-over-6 Ovulation	Coverline Ovulation	LH Test Ovulation	Natural Cycles Ovulation	Logged Libido	Logged Sex
Covariate	*b* (*SE*)	*b* (*SE*)	*b* (*SE*)	*b* (*SE*)	*b* (*SE*)	*b* (*SE*)
Sweden (n = 56,936)						
Intercept	0.0004 (0.160)	0.0002 (0.109)	−7.60 × 10^–8^ (0.209)	0.001 (0.238)	0.757 (0.103)***	0.181 (0.015)***
Age	0.013 (0.003)***	0.005 (0.002)**	1.41 × 10^–6^ (0.004)	0.021 (0.005)***	−0.009 (0.002)***	−0.001 (0.0003)***
BMI Proxy	0.008 (0.003)*	0.004 (0.002)	−2.65 × 10^–6^ (0.001)	0.014 (0.005)**	0.003 (0.002)	3.64 × 10^–4^ (0.0003)
Cycle Length	0.015 (0.003)***	0.005 (0.002)*	6.52 × 10^–7^ (0.004)	0.021 (0.004)***	−0.005 (0.002)**	−0.001 (0.0002)***
Day Length	0.0003 (0.0001)***	0.0004 (0.00004)***	−1.24 × 10^–4^ (0.0001)	0.0002 (0.0001)**	−0.00001 (0.00004)	−6.04 × 10^–7^ (0.000004)
Temperature	0.004 (0.001)***	0.002 (0.001)*	−1.04 × 10^–5^ (0.001)	0.008 (0.002)***	0.001 (0.0006)	2.96 × 10^–4^ (0.0001)***
United States (n = 326,137)						
Intercept	0.000004 (0.069)	0.000003 (0.076)	4.13 × 10^–7^ (0.093)	0.0003 (0.157)	0.680 (0.042)***	0.251 (0.06)***
Age	0.0001 (0.001)	0.0001 (0.001)	2.52 × 10^–5^ (0.002)	0.010 (0.003)***	−0.006 (0.001)***	−0.002 (0.0001)***
BMI Proxy	0.0001 (0.001)	0.0001 (0.001)	9.57 × 10^–6^ (0.001)	0.007 (0.003)*	0.003 (0.001)***	−2.59 × 10^–4^ (0.0001)*
Cycle Length	0.0001 (0.001)	0.0001 (0.001)	2.23 × 10^–5^ (0.002)	0.011 (0.003)***	−0.006 (0.001)***	−0.001 (0.0001)***
Day Length	0.002 (0.0001)***	0.002 (0.0001)***	1.63 × 10^–4^ (0.0001)*	0.005 (0.0001)***	0.0001 (0.00003)***	1.25 × 10^–5^ (0.000004)**
Temperature	0.0001 (0.0004)	0.0002 (0.0004)	1.20 × 10^–5^ (0.001)	−0.003 (0.001)**	−0.0005 (0.0002)*	−8.19 × 10^–6^ (0.00003)

Day Length = number of minutes of ambient day light; BMI = body mass index; *** *p* ≤ .001; ** *p* ≤ .01; *p ≤ .05.

Within the Swedish cohort, using these models yielded no relationship between day length and ovulation measured using Coverline or the Natural Cycles algorithm (*p*s > 0.10). When controlling for temperature, we found that no measure of ovulation—barring 3-over-6 ovulation (*p* ≤ 0.001)—was significantly related to day length (*p*s > 0.30), and day length was no longer related to logged sex or libido (*p*s > 0.40) (Table 6). Restricting the sample to those users most likely to be fertile (i.e., users between the ages of 18 and 45) revealed a positive significant relationship between day length and each measure of ovulation (*p*s ≤ 0.005) with the exception of the LH test measure (similar to what was observed in the original model) but no relationship between day length and logged sex or logged libido (*p*s > 0.10) (Table 7).

As noted, the original results from the US sample replicated with the exception of the LH test results in the model that controlled for temperature. The results of this model again found a significant relationship between day length and ovulation as indicated by LH test results (*p* ≤ 0.01); however, the relationship between these variables became negative, with increased day length being associated with decreased rates of ovulation as indicated by a positive LH test. Potential explanations for these patterns are discussed below.

## Discussion

Many species exhibit seasonal patterns of breeding, with mating effort being highest in the summer months, when resource availability is high and exposure to environmental stressors is low. For example, reproductive activity is highest in the summer for long day breeding species, such as those with short or very long gestation period that breed and birth offspring in the summer. Conversely, species with moderate gestation periods (6–8 months) exhibit increased gonadal function during the fall months so that later stages of gestation and more energetically costly functions, such as lactation, then occur during the summer. Despite the growing body of evidence suggesting seasonal patterns in each testosterone production and conception rates, little is known about the impact of seasonality on human female ovarian function. The results of the present study provide some of the first evidence to date demonstrating that ovarian function may also vary seasonally.

Specifically, the majority of our statistical models found support for the hypothesis that months with longer days were associated with higher rates of ovulation, even when controlling for several individual and personal and environmental factors associated with ovulation in human females (e.g., age, BMI, cycle length) or day length (ambient temperature). Further, the majority our statistical models found support for the relationship between day length and ovulation being one that generalized (a) across samples from two geographically distinct locales (the U.S. and Sweden), particularly when restricting the analyses to include only those users who are likely to be fertile, and (b) across three different ovulation indexes (i.e., ovulation as labeled by the Natural Cycles ovulation detection algorithm, the 3-Over-6 method, or the Coverline method). Lastly, we found no evidence of equinox effects on the relationship between day length and ovulation rate in either sample.

While many of our findings did generalize across both geographic samples, there were some discrepant results that emerged when we attempted to replicate the results using a range-standardized measure of photoperiod. In particular, we found that many of the relationships between day length and women’s ovulation, sexual behavior, and sexual motivation did not reach significance in the Swedish sample when the data was range-standardized (although it is worth noting that the range-standardized results did replicate when run only on the likely-fertile users).

Although unanticipated, there are a variety of factors that could have contributed to this pattern. First, given that the pattern replicated in the Swedish sample when the sample was restricted to women who were most likely to be fertile, it is possible that the relationship between photoperiod and ovulation rate is most evident in younger women due to the greater between-cycle variability that emerges as women age. Second, it is possible that these results may have failed to emerge in response to the relatively narrow range of possible daylight hours *between* individual Swedish women during a given time of year due to Sweden’s relatively small land mass combined with the relatively broad range of possible daylight hours *within* Swedish women across seasons due to Sweden’s relatively large distance from the equator (which grants them far fewer daylight hours in the winter and far more daylight hours in the summer than what is observed in the mainland U.S.). These differences in between- and within- individual variability in day length, particularly when combined with the much-smaller Swedish sample size, may have contributed to the failure of the range standardized models to yield the expect results from the full Swedish sample, as the estimated effects were based on both between and within women effects.

It is additionally possible that the discrepant results between the U.S. and Swedish samples could instead be due to cultural differences interacting with photoperiod effects to impact ovulation rates. For example, research finds that Swedish individuals, on average, maintain a higher quality of life than U.S. individuals^49^. Therefore, it could be that some of the factors contributing to quality of life among Swedish individuals buffer the effects of environmental stressors on women’s fertility. Future research would benefit from examining this possibility.

It is also important to address the discrepancies that arose between models that used LH tests as the marker of ovulation. While our unstandardized models found a positive relationship between day length and the frequency of positive LH tests among U.S. women, the standardized model—as well as both models run using this ovulation diagnostic conducted on the data from the Swedish sample—found the opposite. These contrasting LH results may have emerged in response to LH tests’—despite being a popular method of predicting impending ovulation—high rate of false negatives^8,23^.

It is also possible that these contrasting results could reflect levels of LH being inversely related to the probability of successful ovulation across (but not within) cycles. Much research finds that levels of pituitary releasing hormones (of which LH is one) are often negatively related to receptor density and efficiency^50–52^. If the seasonal changes in ovulation rate are mediated by changes in the efficiency of signaling between LH and ovarian LH receptors (with less efficient signaling occurring in the winter months) this could correspond to both a) increased pituitary release of LH and b) lower rates of successful ovulation. Given that LH tests are often not sensitive enough to reliably detect pre-ovulatory levels of LH, women may be most likely to have a positive LH test during cycles in which the LH-ovarian communication pathway is least likely to result in successful ovulation. This interpretation is consistent with past work demonstrating that high levels of LH are associated with disrupted ovulation in some contexts^53^. Future research would benefit from examining this possibility. Regardless, the LH test measured ovulation results should be interpreted with caution, especially considering the reduced sample size of cycles that had available LH test results.

In addition to the relationship between day length and ovulation rate, we also found evidence of links between day length and women’s self-reported sexual motivation and logged sexual behavior. Women reported having higher libido and logged more sexual behavior on longer days than shorter ones. As with ovulation rate, the sexual motivation and behavior effects in U.S. women remained significant while controlling for variations in relevant individual and environmental factors. However, changes in day length were unrelated to changes in libido among Swedish women. As the libido-specific analyses were restricted to women’s cycles that included at least one logged entry for libido—that is, we exclude cycles which appear to have a libido of 0 due to an absence of logs, rather than logged low libido—the sample sizes for these analyses were reduced to 9,739 cycles (3,854 participants) from Sweden and 68,417 cycles (27,175 participants) from the U.S. This reduced sample size provides one possible explanation of the lack of a significant relationship between day length and logged libido in the Swedish sample. It is also possible that cross-cultural differences in sexual attitudes and behaviors may have contributed to these patterns.

Overall, despite some notable between-location differences, the observed relationships between day length and ovulation rate, sexual motivation, and sexual behavior in the present study are generally consistent with much research in the evolutionary biological sciences that suggest an important role of physical environmental conditions—including day length—in influencing how organisms regulate investment in reproductive activity^38,54^ and adapt trade-offs in somatic maintenance versus reproduction^55,56^. Together, these results suggest that human physiological systems—including the functioning of women’s HPG axes—may be seasonally sensitive. That these patterns continue to persist despite the numerous modifications to our environment to minimize the experienced impact of seasonal changes (e.g., temperature-controlled homes, artificial light sources, etc.), is a testament to their embeddedness in our biology. Taking these differences into account could play an important role in shaping the future of contraception, fertility planning, and fertility treatments, as the success of each of these outcomes is likely to be touched by the observed seasonal patterns in reproductive function.

Along with differences in between-location relationships, there are several limitations of the present study that should be considered. First, there are theoretical limitations. Specifically, we are unable to determine whether the observed relationship between day length and ovulation rate has emerged in response to *greater* investment in reproductive function during the summer months or *decreased* investment in reproductive function during the winter months. Given past research finds increased investment in immunological activity and concomitant reductions in total levels of the sex hormone testosterone in men during shorter days^12^, the effects observed in the current results may be (a) driven by adaptations designed to promote reproduction in the spring and summer months or (b) a byproduct of increased investment in bodily maintenance and repair during the fall and winter months. Future work would benefit from identifying the mechanisms that underlie these seasonal patterns to better understand what, if any, adaptive function they might serve.

Additionally, although the present study was able to rule out ambient temperature as an alternative driver of the observed relationship between day length and female reproductive function, temperature is but one of many possible environmental factors that vary alongside seasonal shifts in biological and behavioral functioning. Future research would therefore also benefit from collecting a broader range of environmental measures that have been implicated in human seasonality research, such as vacations and time-off of work. Lastly, the present research does not experimentally assess the biological mechanisms driving fluctuations in ovulation rate as a function of day length, which provides an important area for future research. One such biological mechanism that could present a promising direction for future research is the role of pineal melatonin. Pineal melatonin secretion is inversely proportional to day length duration^1,57^, and is implicated in the seasonal reproductive functioning of several non-human animals^21,58^.

### Conclusions

Through examination of longitudinal data from two large samples across two countries, the present research finds that human females’ ovarian function may be related to day length, with longer days being associated with higher ovulation rates. The present results also found complementary shifts in women’s sexual motivation and behavior, with women logging higher libido and more sexual behavior during times in the year with longer days relative to shorter. These results are consistent with a growing body of work demonstrating powerful seasonal patterns in human biological systems, many of which can have implications for everything ranging from healthcare to population planning.

## Method

### Natural cycles health application

Data were obtained from users of Natural Cycles, an FDA cleared birth control app. The app uses a proprietary statistical algorithm that estimates the probability of conception on a given day. Users are recommended to take their temperature 5 times per week before getting out of bed first thing after waking up. They input this temperature into the app before it gives a daily fertility status. There are three modes in the app, NC° Birth Control, NC° Plan Pregnancy, and NC° Follow Pregnancy, allowing users to choose the most suitable option for their current intentions. Most women start using the app in NC° Birth Control, that is, to prevent a pregnancy by avoiding unprotected intercourse during days which the app calculates to be fertile days. Users may subsequently change their intentions and are instructed to register this by switching to NC° Plan Pregnancy, in which the app serves as an aid to the timing of intercourse to achieve conception.

The core of accurately detecting ovulation is the same for both products, but there are differences in the risk profile. For NC° Birth Control, the algorithm is optimized to have a very low risk of wrongly attributing a green (safe) day in the fertile window, while for NC° Plan Pregnancy, it is optimized to better isolate the fertile window and the most fertile days. The same colors are displayed (red for fertile and green for not fertile); however, in NC° Birth Control a day is either strictly red or green, whereas in NC° Plan Pregnancy a color scale is used during the fertile window.

The Natural Cycles algorithm identifies ovulation retrospectively based on the first day of menstruation and basal body temperatures, which may be supplemented by positive urinary LH tests. Basal body temperatures are recorded each morning using a thermometer sensitive to the hundredth place, and with measures excluded if the user reports any illness, alcohol intake, or changes in sleep that might influence basal temperatures. To reduce the risk of misidentifying ovulations, the algorithm reports ovulation by rising basal body temperature only if the average temperature from three consecutive calendar days is greater than the woman’s follicular phase average and her baseline average across all data entries, as well as consistent with her luteal phase average. If no temperature rise is observed and the data quality and quantity is deemed sufficient, the cycle is flagged as anovulatory.

### Participants

Participants included an initial sample of 293,224 women between the ages of 18 and 63 years old with approximately 1.7 million logged cycles on the Natural Cycles application. Participants provided informed consent for the use of their data in research during registration. The Texas Christian University Institutional Review Board has waived the need for ethical approval in this study (IRB-2022-459). All experiments were performed in accordance with the relevant guidelines and regulations. Participants in this initial sample included women who logged at least one menstrual cycle and declared no ovulation-disrupting preconditions (i.e., PCOS, endometriosis, menopause, or thyroid-related conditions). We excluded data from cycles that were greater than 40 days in length and cycles in which women logged fewer than 10 basal body temperature measurements. For participants who reported a miscarriage, pregnancy, or taking an emergency contraceptive, we excluded the cycle in which the event was recorded and the cycle immediately following. If a pregnancy is reported, we excluded the two cycles immediately following the pregnancy.

Next, we isolated data from the two largest geographical cohorts with regional information present in the sample, which included women living in each Sweden and the United States. These cohorts served as our data analytic sample. The final sample included approximately 65,240 women with 353,411 reported cycles from the United States and 10,940 women with 72,498 reported cycles from Sweden.

From each of these samples, data from women were excluded if they lacked sufficient geographical specificity to assess day length. This included women from the following regions: AP (Armed Forces Pacific), AE (Armed Forces Europe), MH (Marshall Islands), AA (Armed Forces America), or lack of region recognition (FM and BY). This exclusionary criterion only affected women from the U.S. cohort, leading to the removal of 9 participants’ data. After this exclusion, The US sample included 65,231 women reporting 353,351 cycles. We also exclude cycles for which we have no temperature data, to ensure consistency in the cohorts used for each model. This leaves 61,696 participants and 326,137 cycles in the US data and 8,678 participants and 56,936 cycles in the Swedish data. Sample characteristics can be found in Table 1. The maximum number of cycles reported by a single participant is 13 and the minimum is 1.

### Measures

#### Measures of ovulation rate

Ovulation rate was assessed using basal body temperature (BBT) based ovulation detection algorithms (confirmatory measures of ovulation) and luteinizing hormone (LH) tests. Basal body temperature increases slightly—typically less than a 1/2 degree F (0.3 C)—when ovulation occurs. Ovulation is confirmed using BBT when the slightly higher temperature remains steady for three days or more. In this way, BBT-based measures are confirmatory, as they label a cycle as ovulatory based on physiological changes that occur in the body as a consequence of ovulation. LH tests, on the other hand, are anticipatory, as these tests measures levels of luteinizing hormone in the urine, which only reach detectable levels prior to ovulation, when pituitary release of LH surges to prompt ovarian release of the now mature ovum.

To measure participants’ ovulation rate, we used 4 measures, including two established basal body temperature-based ovulation prediction algorithms (the Coverline and 3-over-6 methods), one using a proprietary basal body temperature-based ovulation prediction algorithm (the Natural Cycles ovulation detection algorithm), and a final measure assessing ovulation based on hormonal measurements (LH tests). The Coverline method^59^ operates by examining the first 10 days of a participant’s menstrual cycle. The ‘coverline’ is then calculated by taking the maximum basal body temperature measurement from the first 10 days, adding to it 0.15 degrees (Fahrenheit), and then identifying if any subsequent temperature exceeds this threshold. If so, the cycle is declared ovulatory. The 3-over-6 method^60^ declares a cycle as ovulatory based on the presence of three consecutive basal body temperature measurements that exceed the maximum of the preceding six temperature measurements during the same cycle. We also used the Natural Cycles ovulation label and LH test results (when available) as robustness checks. The Natural Cycles proprietary algorithm makes use of prior logged data from the Natural Cycles application to determine whether ovulation occurred. LH tests detect a spike in luteinizing hormone, which precedes ovulation and were recorded for 31% of available cycles.

#### Day length

Day length was operationalized as the time elapsed between sunrise and sunset. These data were imported from the Suntime library^61^ based on participants’ geographical coordinates. Geographic coordinates for day length measurement were assessed using the central latitude and longitude of a participant’s reported county (Sweden) or state (United States). For example, all participants in the state of California were assigned the geographic coordinates (latitude = 33.944197^°^, longitude = -118.402446^°^), participants in the state of Massachusetts were assigned the geographic coordinates (latitude = 42.4072^°^, longitude = 71.3824^°^), and so on. By assuming each participant is at the state or county’s center, we introduce a day length estimation error of up to 25 min. Note that this corresponds studying the impact of absolute day length, rather than relative day length (i.e. days growing longer or shorter).

#### Daytime temperature

While day length is a powerful predictor of upcoming seasonal changes for a wide variety of species^62–64^, including humans (e.g. ^12^,), it is possible that it is not the preeminent seasonally-variable cue that modulates women’s ovarian function (e.g. ^34^,). For example, given that temperature extremes can initiate physiological stress responses (see^65^ for review), which can disrupt ovulation^66^, it is possible that the predicted relationship between day length and ovulation rate may occur as a byproduct of each of these variables being linked to temperature. To test for this alternative account of the predicted results, we used the Meteostat library^67^ to import data on location-specific temperature for each participant using the same latitude and longitude estimate described above. Temperatures were imported for the start date for each reported cycle. We exclude cycles where Meteostat does not offer the temperature (this occurs for ~ 10% of the cycles).

#### Logged libido and logged sex

On each cycle day, participants have the opportunity to log their libido and sexual behavior within the app. Options for libido are: low (1), medium (2), or high (3). Options for logging sexual behavior are: had unprotected sex, had protected sex, or had no sex.

All logged sexual behaviors (“had sex” and “had unprotected sex”) were first collapsed into a single variable “had sex”. Next, on days in which participants did not log libido or sexual behavior, the missing data was assigned a score of 0. To calculate each cycle’s average libido and sexual behavior, we then dropped all days with a score of zero to ensure that our estimates of libido and / or sexual behavior were not artificially low for those participants who did not log frequently. We then took the average of each reported libido and sexual behavior score from the data logged across the remaining days. For example, if a participant has logged sexual activity on 3 of 4 daily logs in a given cycle, this cycle would receive a sexual activity score of 0.75.

### Data analysis plan

Prior to analyzing the data, we examined each covariate (age, body mass index [BMI], cycle length, and day length) for normality and found each to be approximately normal and need no transformation. A large majority of participants’ daily logs did not record libido or sexual activity (96% and 82% respectively). Consequently, imputation was not feasible and cycle aggregate measures were computed for each behavioral measure, instead.

It is rare for a participant to log BBT during each day of the data collection period. Since most temperature-based ovulation classifiers require sequences of recorded temperatures, we estimate missing BBT measurements using data imputation. To do this, we will use an approach called cubic spline-fitting, which allows estimation of a continuous curve from a set of points. Cubic spline-fitting is an appropriate interpolation method as, (1) it is robust to uncertain measurements (logged BBT can be irregular due to the time of measurement and participant behavior, including diet, exercise, and sleep) and (2) this approach demonstrates lower error than other polynomial-based fitting procedures. Further, this approach has also been supported by recent literature examining BBT imputation (see e.g. ^63^,). Concisely, cubic spline-fitting fits a set of piecewise cubic functions to the observed data to produce a twice-differentiable estimate of the true function (in this case, a function that maps cycle day to temperature). The method estimates the piecewise cubic-function through a system of equations derived from the differentiability constraints (for example, that the second derivatives at boundary points between adjacent pieces of the function are equivalent). Differentiability constraints are important to ensuring that the curve is smooth through all observed measurements.

We will use generalized logistic mixed effects models to test our predictions. This model was chosen to adjust for the multilevel nature of the data (i.e., cycles nested within participants), and to account for the binary nature of the outcome variable (i.e., did or did not ovulate at each cycle). Random intercepts will be added per participant to control for the nested data structure. We considered random slopes per participant and found that the model failed to converge due to missing data. Model parameters will be found using the GPBoost software^68^ and we will report beta coefficients, *p*-values, and standard errors to demonstrate the robustness of the analysis.

In each analysis, we will adjust for three covariates with established relationships to ovulation: age, BMI, and cycle length. Follow-up analyses will be performed to identify whether the relationship between day length and each ovulation, logged libido, and sexual behavior persist while controlling for seasonal differences in ambient temperature. We then restrict our focus to participants aged 18–45 years old, who are most likely fertile, to examine the stability of our findings. Additionally, we will examine the robustness of our findings to covariate normalization by including day length in units of half-hours, where the covariate mimics the ranges of BMI (up to 56), age (up to 63), and cycle length (up to 40 days). Finally, because others have found evidence that human conception rates peak near the spring equinox^45^, we will test for the presence of equinox effects on each ovulation, logged libido, and sexual behavior. Each of the above analyses will be considered with respect to all 4 methods of ovulation rate measurement: the Natural Cycles method, the Coverline method, the 3-over-6 method, and LH tests.

## Data Availability

Data that support this study are available from the corresponding author (D.S.) subject to data access approval from Natural Cycles. Due to the sensitivity of the underlying data, the data cannot be made public.
